# Dual Corneal-Graft Rejection after mRNA Vaccine (BNT162b2) for COVID-19 during the First Six Months of Follow-Up: Case Report, State of the Art and Ethical Concerns

**DOI:** 10.3390/vaccines9111274

**Published:** 2021-11-03

**Authors:** Matteo Nioi, Ernesto d’Aloja, Maurizio Fossarello, Pietro Emanuele Napoli

**Affiliations:** 1 Forensic Medicine Unit, Department of Medical Sciences and Public Health, University of Cagliari, 09040 Cagliari, Italy; ernesto.daloja@unica.it; 2 Department of Surgical Sciences, Eye Clinic, University of Cagliari, 09124 Cagliari, Italy; mfossarello@aoucagliari.it

**Keywords:** COVID-19, BNT162b2 vaccine, BNT162b2 vaccine side effect, COVID-19 vaccines corneal graft, COVID-19 vaccines transplant rejection, COVID-19 vaccines and corneal graft rejection, COVID-19 immunomodulation, COVID-19 vaccines side effects, COVID-19 vaccines immunomodulation, COVID-19 vaccines vitamin D

## Abstract

Present mass vaccination against Coronavirus Disease-19 (COVID-19) is the most widely used health policy and the most promising approach to curb the severe acute respiratory syndrome coronavirus 2 (SARS-CoV-2) pandemic globally. However, new side effects are emerging from the mass vaccination not described during the experimental stages. In the present study, we discuss a case of acute corneal graft rejection, which has occurred 25 years after transplantation and 13 days after the administration of the BNT162b2 vaccine (Comirnaty, BioNTech/Pfizer), which was followed-up for a period of six months. In this period, the corneal inflammation appeared twice but was successfully managed with topical therapy and supplementation of Vitamin D. A risk of corneal graft rejection must be included in the list of potential vaccine complications, in order to inform the transplanted patient to undergo a preliminary and a follow-up ocular examination, and eventually to include corneal graft in the list of contraindications to vaccination.

## 1. Introduction

More than fifteen months after the outbreak of Coronavirus Disease-19 (COVID-19) pandemic, vaccines appear to be a promising approach for the resolution of the infection [1,2]. However, after the Food and Drug Administration (FDA) and the European Medicines Agency (EMA) granted the “emergency use authorization” of the COVID-19 vaccines developed by BioNTech/Pfizer and Moderna, numerous debates sprung up within the scientific community regarding the safety of the post-marketing phase, and side effects not described during the experimental stages [3,4,5]. A detailed description regarding the complications from the COVID-19 vaccines allows physicians, scholars, and patients to make a correct estimate of the risk–benefit ratio of vaccination and address the side effects immediately as they appear.

Recently, seven cases of both corneal lamellar and penetrating graft rejection following immunization against SARS-Cov-2 [6,7,8,9,10] (Table 1) have been described.

Although many studies confirm that the human eye represents a dual route of transmission for SARS-CoV-2, which is based on the presence of ACE2 receptors on the ocular surface, the phenomenon of corneal transplant rejection following vaccine administration is not completely understood [11,12,13,14]. Actually, several studies have also described cases of corneal graft rejection following the administration of influenza vaccines or yellow fever vaccines [15,16,17,18,19,20].

## 2. Materials and Methods

In the study, we described a single case observed and treated at the Eye Clinic of University of Cagliari, (San Giovanni di Dio Hospital).

### 2.1. Clinical and Laboratory Assessment

The patient performed the clinical and laboratory exams as follows: complete ophthalmological visit (visual acuity, intraocular pressure, slit-lamp biomicroscopy, fundus oculi), sampling of anterior chamber (AC) aqueous humor for reverse transcriptase-polymerase chain reaction (RT-PCR), complete blood count and vitamin D assay.

### 2.2. Optical Coherence Tomography (OCT) Imaging

Anterior OCT imaging was performed with Cirrus HD-OCT 4000; Carl Zeiss Meditec, Inc., Dublin, CA, USA. This device is a Fourier-domain OCT platform that has a 5 µm axial resolution, takes 27,000 axial scans per second, and works at a wavelength of 840 nm. The cross-sectional corneal images were obtained using the Anterior Segment 5 Line Raster scanning protocol (a set of five parallel lines of equal length at 3 mm). Each scan line comprised 4096 A-scans.

This protocol was designed to identify alterations in the Anterior Segment, such as inflammatory floaters in AC (cells or proteins detectable in situations of corneal tissue inflammation, e.g., acute rejection).

### 2.3. Informed Consent and Independent Review Board

Written informed consent to publish this case report and the accompanying images were obtained from the patient.

Since only normal clinical practice is described in this case report, formal ethical approval by the Independent Review Board was not required in accordance with the policy of our Institution.

## 3. Case Report

A 44-year-old Caucasian woman presented with a 2-day history of blurred vision, redness, and discomfort in her left eye, 13 days after receiving the first shot of the SARS-CoV-2 mRNA vaccine BNT162b2 (Comirnaty, Pfizer-BioNTech, Mainz, Germany). Systemic reactions were absent. The family anamnesis was unremarkable. The patient was unemployed. Medical history was unremarkable. Ocular history included penetrating keratoplasty (PK) performed 25 years before in her left eye due to keratoconus.

**Figure 1:**
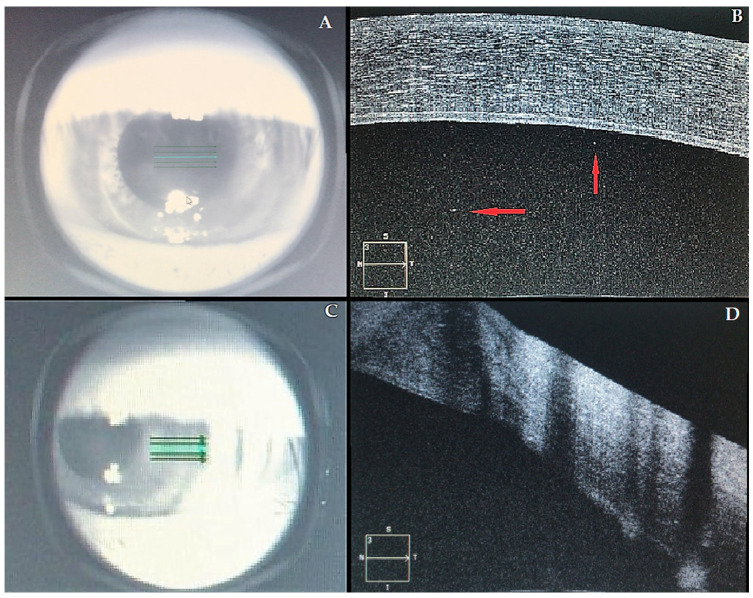
Acute corneal graft. (**A**–**C**) Infrared images of the left eye; (**B**–**D**) correspondent OCT images. Central cornea (**A**,**B**): in Figure 1B, it is possible to observe a marked corneal edema and a series of morphological changes (see text). Red arrows indicate a number of inflammatory floaters in the anterior chambre. Transition area (**C**,**D**) between the recipient’s cornea (normal thickness) and the graft (thickened by edema).

At presentation, her left best-corrected visual acuity (BCVA) was counting fingers, compared to 20/30 recorded six months before. Intraocular pressure was 12 mm Hg. Slit-lamp biomicroscopy revealed ciliary injection, diffuse corneal edema within the graft, keratic precipitates, Descemet folds, and anterior chamber (AC) cells were noted, indicating corneal graft rejection. The right eye was unremarkable. Fundus examination was normal in both eyes. Anterior segment OCT confirmed a series of morphological changes (corneal thickening, subepithelial bullae, internal cornea folds, hyper-reflective points in AC, irregularities of the endothelium). Central corneal thickness (CCT) was 692 μm, compared to 560 μm of a previous visit (Figure 1). An AC aqueous sample was examined by polymerase chain reaction (PCR), and it was negative for cytomegalovirus, herpes simplex virus, and varicella-zoster virus. The complete blood count was normal. However, a severe vitamin D deficiency (circulating 25-hydroxyvitamin D [25(OH)D] concentration of 9 ng/mL) was detected. (Table 2).

Treatment was initiated using hourly steroid drops, i.e., dexamethasone 0.2% (LUXAZONE^®^, Allergan, Rome, Italy). In addition, a vitamin D supplement (cholecalciferol, DIBASE^®^, Abiogen Pharma, Ospedaletto, Italy) was prescribed (daily intakes of 1000 IU of vitamin D for 15 days). At 1-week follow-up, corneal clouding appeared improved, visual acuity was 20/100, corneal edema was reduced (CCT = 597 μm), and only rare keratic precipitates and AC cells were visible. Therefore, topical steroid treatment was slowly tapered. Two weeks later, further improvement of the corneal edema was revealed (CCT = 578 μm), and the visual acuity returned to baseline. Four weeks after the onset of corneal graft rejection, no active inflammation (CCT = 562 μm) was detected, and the visual acuity remained 20/30.

At a 4-week follow-up, a further episode of rejection in her left eye was observed, in parallel with persistence of vitamin D deficiency (serum [25(OH)D] of 19 ng/mL). The corneal clinical picture was similar to previous episode. Steroid drops were re-started, and higher doses of vitamin D were prescribed (50,000 IU of vitamin D three times weekly). The corneal inflammation regressed in four weeks, and at six-months of follow-up, no further rejection episodes were observed. Nonetheless, in agreement with her GP, the patient refused to receive the second dose of the vaccine.

## 4. Discussion

The current report describes a case of biphasic acute corneal allograft rejection following a penetrating keratoplasty (PK) performed twenty-five years earlier, occurring approximately two weeks after the BNT162b2 (Comirnaty, BioNTech/Pfizer, Mainz, Germany ) vaccine administration. To our knowledge, this is the first case report regarding a similar adverse event that evaluates a follow-up period of six months in parallel with a vitamin D blood assay.

Overall, of the seven cases reported of corneal rejection following COVID-19 vaccination so far in the literature, the most frequently reported corneal transplant types were Perforating Keratoplasty (PK) and Descemet Membrane Endothelial Keratoplasty (DMEK) (42.9% and 42.9% of cases, respectively), while Descemet Stripping Automated Endothelial Keratoplasty (DSAEK) was found only in 14.2% of cases. Five of the seven individuals affected were men (71.4%) and two were women (28.6%). The mean age was 68 ± 8.1 years. The vaccine most frequently associated with corneal rejection was the BNT162b2 (85.8% of cases). The adverse event developed on average at 14.8 ± 12.3 months after surgery. The average interval between the execution of the vaccine and the occurrence of rejection was 14.9 ± 8.8 days. In 100% of cases, the therapy consisted of ocular steroids drops (TS). In three of seven cases (42.9%), corticosteroids therapy with oral prednisone (OP) was associated with the TS; in one case (14.2%), Acyclovir was associated with TS. In six cases (85.8%), there was a complete recovery; in one case (14.2%), the outcome is unknown.

### 4.1. Causality

Although it is difficult to demonstrate the causality between COVID-19 vaccine and corneal graft rejection, our case shows a temporal association between the two events. Moreover, a series of issues and criteria described in the specific guidelines (Causality Assessment of an Adverse Event Following Immunization—AEFI) suggests that this rare adverse event is likely due to immunization through a number of pathophysiological pathways [21].

From the first level of analysis (i.e., the evaluation of possible factors supporting the causal association), it is of particular importance the local immune tolerance (i.e., the relative immune privilege to the cornea from anterior chamber–associated immune deviation—ACAID) that has been maintained for 25 years after surgery. Moreover, considering the excellent state of health of the patient, we can exclude other possible etiological factors responsible for the rejection.

The second level of analysis concerns the description and the assessment of the casual association reported in the *medical literature*. Although there are a number of case reports that suggest a plausible biological relationship between the administration of the vaccine and the occurrence of the event, no high-level scientific evidence exists to confirm this relationship (e.g., meta-analyses) [6,7,8,9,10].

The third level of analysis regards the possibility that the event occurs within the *time window* of increased risk after a plausible cause. The thirteen-day lag between vaccine administration and corneal graft rejection that we observed falls within the window of increased risk described in the specific guidelines (AEFI) [21].

The fourth level of analysis (i.e., the evaluation of possible factors *against* a causal association) requires the proof of the absence of strong elements against a relationship between COVID-19 vaccination and the adverse event. Concerning this criterion, no strong elements indicate the absence of a causal link.

At the fifth level of analysis, AEFI guidelines recommend to evaluate other “qualifying factors” (i.e., previous similar episodes with or without vaccination, or exposure to drugs/allergens/over-the-counter products—OTCs). In our case report, no “qualifying factors” may be responsible for the rejection. In fact, after surgery for keratoconus, the patient had no other pathologies in progress (no drugs/no OTCs). Moreover, the patient had no previous reactions to vaccines, nor exposure to toxic substances.

### 4.2. Possible Pathogenetic Mechanisms

Although the mechanisms that lead to corneal transplant rejection have not been fully understood, the dysregulation of the immune system probably plays a crucial role, regardless of the genetic predisposition [22,23,24,25,26].

Phylactou et al. hypothesized several mechanisms concerning the pathogenesis of the allograft rejection. The first mechanism envisages the activation of the immune system following the vaccine, involving the occurrence of a direct allorecognition presumably by a direct pathway. The second possible mechanism predicts that antibodies play a central role in the dynamics of rejection (cross-reaction) [6]. Accordingly, COVID-19 vaccines have been shown to induce SARS-CoV-2 neutralizing antibodies and elicit strong Th1-biased CD4+ responses in humans. CD4+ Th1 cells have been shown to be the main mediators of corneal graft rejection [10]. Overall, corneal rejections after immunization are not a new phenomenon. An article of 1988 by Steinemann analyzed the problem of corneal transplant rejection after immunization. Among the etiological mechanisms hypothesized was local inflammation owing to the deposition of immune complexes, the pro-inflammatory action of interferon-gamma released at the systemic level, and that immunization may stimulate the production of T effector cells that cross-react with allo-major histocompatibility complex antigens of the corneal button [27]. Moreover, several immune-mediated reactions seem to determine other side effects of mRNA vaccines such as myocarditis, thrombocytopenia, and herpes zoster infection [28].

Future studies are required to enhance a greater understanding of these hypotheses concerning the COVID-19 mRNA vaccines.

Moreover, in our case, low values of vitamin D (which is an important fat-soluble secosteroid) may have facilitated and supported the adverse reaction.

The importance of verifying an adequate plasma level of vitamin D in transplant recipients is supported by an abundant and consolidated literature [29,30,31,32,33,34]. The molecular mechanism by which vitamin D inhibits transplant reaction is related to its immunomodulatory proprieties. In fact, cholecalciferol can decrease the expression of IL-2 and interferon-mRNA, as well as reduce the proliferation and cytotoxic activity of T cells (CD4^+^ and CD8^+^). Moreover, vitamin D can suppress the expression of the major histocompatibility complexes of classes II, CD40, CD80, and CD86, thus decreasing differentiation, maturation, and immunostimulating capacity of dendritic cells that constitutively express the cholecalciferol Receptor and CYP27B1 [35,36,37,38,39,40].

Accordingly, the implementation of this vitamin was temporarily associated with the resolution of the corneal graft reject and the absence of further recurrences. This suggests the importance of monitoring vitamin D levels in the peri-vaccination period and in case of corneal transplant rejection.

### 4.3. Peculiarities of the Case and Ethical Conundrum

Some peculiarities in our case are consistent with those presented in the literature [6,7,8,9,10].

Firstly, this is primarily due to the time span between the transplants and the time of rejection (approximately 25 years).

Secondly, in comparison with other cases, our case describes a six-month follow-up period. This observation period has shown that, even after an initial resolution of the rejection, new episodes may arise that must be intercepted and treated early. These data are of absolute importance because they raise serious questions about the risk–benefit ratio of the second dose of vaccine after rejection following the first administration (in fact, the patient refused to receive the second dose of the vaccine). This point opens important ethical concerns that can only be addressed considering the deeper aspects of the immunologic mechanisms of rejection. Moreover, the administration of oral steroids can also represent an ethical dilemma as it reduces the ability of the immune system to produce an adequate level of antibodies against COVID-19 in a patient who is likely not to receive the second dose.

Thirdly, corneal graft rejection is described in our work for the first time after administration of COVID-19 vaccine with concomitant vitamin D deficiency. The latter clearly represents an important clue for immunological dysregulation (i.e., maladaptive changes in molecular control of immune system processes) that may have a fundamental role in the corneal rejection.

### 4.4. Practical Implications

This report suggests that, before vaccination, is of fundamental importance to collect a complete medical-history of the patient and to inform the latter regarding all the potential adverse effects of this injection. Thus, our case adds a piece to the puzzle of ocular adverse reactions from COVID-19 vaccines, and encourages the execution of laboratory/ophthalmological exams (e.g., serum levels of vitamin D), as well as a close follow-up after immunization. Clearly, a patient aware of this possible adverse event may be more inclined to check his/her medical condition. Considering the widespread use of COVID-19 vaccination, it is likely that further cases of corneal graft rejection will be reported in the next future [41,42].

If the causal relationship between corneal transplant rejection and COVID-19 vaccination will be confirmed in the scientific literature, the estimation of the risk/benefit ratio should be provided during the administration of the consent in order not to damage the patient’s right to self-determination. In some countries, the doctor’s failure to inform the patient about possible side effects of the vaccine can have medico-legal consequences [43].

Some authors hypothesized that, when rejection occurs, the administration of steroids through the local or oral route before the vaccine administration could be useful for preventive purposes [18]. Considering the pathogenesis of corneal graft rejection and the immunomodulatory effects of vitamin D deficiency in transplanted individuals, preventive check and integration of this vitamin could be considered before the vaccine administration.

## 5. Conclusions

Although corneal graft rejection after COVID-19 vaccination is still poorly understood, our case underlines the importance of a close follow-up after the onset of symptoms in these patients and a possible pathogenesis owing to an immune system dysregulation related to vitamin D deficiency. Future studies are needed to elucidate the mechanisms underlying potential transplant failure after immunization against SARS-CoV-2 and to clarify the epidemiological features of this complication.

## Figures and Tables

**Table 1:** Cases of corneal graft rejection after COVID-19 vaccination. COT-INT Cause of transplantation- Intervention; IIR: Interval intervention-rejection; IVR: Interval vaccine- rejection; F: female; M: male; CPB: Comirnaty, BNT162b2, BioNTech/Pfizer; DMEK: Descemet Membrane Endothelial Keratoplasty; FECD: Fuchs Endothelial Corneal Dystrophy; I°D: first dose; II°D: second dose; TS: topical steroid; DEX: Dexamethasone; PK: penetrating keratoplasty; KC: keratoconus; RG: regraft; OP: oral prednisone; CCS: childhood corneal scar; CHA: ChAdO × 1 nCoV-19 Corona Virus Vaccine Recombinant COVISHIELD™, DSAEK: Descemet Stripping Automated Endothelial Keratoplasty; fDAEK: failed Descemet Stripping Automated Endothelial Keratoplasty; ACV: acyclovir.

Study	N	COT-INT	Sex	Age	Vaccine	IIR	IVR	Therapy	Outcome
**Phylactou et al., 2021 [6]**	2	DMEK for FECD	F	66	CPB	14 days	7 days (I°D)	TS (DEX)	Full recovery
		DMEK and cataract surgery for FECD	F	83	CPB	3 years	2 months (I°D) 3 weeks (II°D)	TS (DEX)	Full recovery
**Wasser et al., 2021 [7]**	2	PK for KC RG	M	73	CPB	2 years	13 days (I°D)	TS (DEX) + OP	Full recovery
		PK for KC	M	56	CPB	10 months	14 days (I°D)	TS (DEX) + OP	Full recovery
**Ravichandran et al., 2021 [8]**	1	PK for CCS	M	62	CHA	2 years	3 weeks (I°D)	TS	Unknown
**Crnej et al., 2021 [9]**	1	DMEK	M	71	CPB	5 months	7 days (I°D)	TS + OP	Full recovery
**K.I. Rallis et al., 2021 [10]**	1	DSAEK for FECD; PK for fDSAEK	M	68	CBP	4 months	4 Days (I°D)	TS + ACV	Full recovery

**Table 2:** Blood exams of the patient. Abnormal data are indicated in bold.

Blood Component	Abbreviation Used	Reference Range	SI Reference Range
White blood cells	WBC	4.8 × 10^9^/L	4.5–11.0 × 10^9^/L
Red blood cells	RBC	3.7 × 10^12^/L	Female: 3.5–5.5 × 10^12^/L
Hemoglobin	HGB	1.93 mmol/L	Female: 1.86–2.48 mmol/L
Hematocrit	HT	0.40	Female: 0.36–0.46
Mean corpuscular volume	MCV	93 fl	80–100 fl
Mean corpuscular hemoglobin	MCH	0.45 fmol/cell	0.39–0.54 fmol/cell
Mean corpuscular hemoglobin concentration	MCHC	4.97 mmol Hb/L	4.81–5.58 mmol Hb/L
Platelets	Platelets	239 × 10^9^/L	150–400 × 10^9^/L
**Metabolic And Electrolyte Panel**			
Blood urea nitrogen	BUN	10 mg/dL	6–20 mg/dL
Creatinine	Cre	1.0 mg/dL	Female: 0.6–1.1 mg/dL
Glucose	Glu	89 mg/dL	70–99 mg/dL
Calcium	Ca+	8.6 mg/dL	8.6–10.2 mg/dL
Sodium	Na+	140 mEq/L	136–145 mEq/L
Potassium	K+	4.2 mEq/L	3.5–5.1 mEq/L
Chloride	Cl-	101 m Eq/L	98–107 mEq/L
**25-hydroxyvitamin D [25(OH)D]**	**Vit. D**	**9 ng/ml**	**20–40 ng/ml**
**RT-PCR**			
**Exam**	**Result**		
Acqueous IgG/IgM cytomegalovirus (CMV)	*Negative*		
Acqueous IgG/IgM herpes simplex virus (HSV)	*Negative*		
Acqueous IgG/IgM varicella-zoster virus (VZV)	*Negative*		

## Data Availability

The data that support the findings of this study, after adequate anonymization that protects the patient’s privacy, will be available on request from the corresponding authors (M.N. and P.E.N.). The data are not publicly available due to them containing information that could compromise research participant privacy/consent.
